# Bidirectional Longitudinal Study of Frailty and Depressive Symptoms Among Older Chinese Adults

**DOI:** 10.3389/fnagi.2022.791971

**Published:** 2022-02-10

**Authors:** Limin Cao, Yuhan Zhou, Huiyuan Liu, Mengyuan Shi, Yingliang Wei, Yang Xia

**Affiliations:** ^1^The Third Central Hospital of Tianjin, Tianjin, China; ^2^Department of Clinical Epidemiology, Shengjing Hospital of China Medical University, Shenyang, China; ^3^Department of Orthopedics, Shengjing Hospital of China Medical University, Shenyang, China

**Keywords:** depressive symptoms, frailty, bidirectional association, China, older people

## Abstract

**Objective:**

Frailty and depression, as two common conditions among older adults in China, have been shown to be closely related to each other. The aim of this study was to investigate the bidirectional effects between frailty and depressive symptoms in Chinese population.

**Methods:**

The bidirectional effect of frailty with depressive symptoms was analyzed among 5,303 adults ≥ 60 years of age from the China Health and Retirement Longitudinal Study (CHARLS). Phenotype and a frailty index were used to measure frailty. Depressive symptoms were evaluated using the Epidemiological Studies Depression Scale (CES-D). Logistic regression and Cox proportional hazard regression models were used to determine the bidirectional effects of frailty and depressive symptoms in cross-sectional and cohort studies, respectively. Subgroup and sensitivity analyses were further used to further verify the associations.

**Results:**

In the cross-sectional study, the multivariate-adjusted ORs (95% CIs) for depressive symptoms among pre-frail and frail adults, as defined by the frailty index and phenotype, were 3.05 (2.68–3.49), and 9.78 (8.02–12.03), respectively. Depressed participants showed higher risks of pre-frailty and frailty [frailty index, 3.07 (2.69–3.50); and phenotypic frailty, 9.95 (8.15–12.24)]. During follow-up, the multivariate-adjusted HRs (95% CIs) for depressive symptoms among pre-frail and frail participants, as defined by the frailty index and phenotype, were 1.38 (1.22–1.57), and 1.30 (1.14–1.48), respectively. No significant relationship existed between baseline depressive symptoms and the incidence of frailty. Moreover, the results from subgroup and sensitivity analyses were consistent with the main results.

**Conclusion:**

Although a cross-sectional bidirectional association between depressive symptom and frailty has been observed in older (≥60 years old) Chinese adults, frailty may be an independent predictor for subsequent depression. Moreover, no effect of depressive symptoms on subsequent frailty was detected. Additional bidirectional studies are warranted in China.

## Introduction

The aging population is a global phenomenon, with 1 billion adults ≥ 60 years of age. This number is estimated to reach 1.4 billion by 2030, and will be on the rise in the ensuing decades (World Health Organization, 2018, 2019). The old adults are more likely to have age-related disorders, including mental health and physical fitness, given the limited regenerative abilities. Moreover, the old adults are more likely to have more than one co-morbidity at the same time (World Health Organization, 2019). Indeed, depression, a common mental illness, has a prevalence ranging from 10 to 20% in the older population (Rodda et al., 2011). In addition, depression is associated with a 6–10%, and 30% rate of disability in the primary care setting, and in medical and long-term care settings, respectively (Reynolds et al., 2012). Frailty is a common physical condition among older adults, and is characterized by a functional decline in multiple physiologic systems that causes an increased susceptibility to stressors (Hoogendijk et al., 2019). As reported, the prevalence of frailty has been estimated to range from 10% among community-dwellers (Collard et al., 2012) to 18–40% in hospitalized patients (Cunha et al., 2019). When stressors, such as acute illness, occur, a person with frailty rapidly has a deterioration in functional capacity. Generally, several instruments have been established to identify frailty. The two most common frailty instruments used in studies and clinical settings are the phenotypic and the frailty indices. The phenotype model of frailty considers frailty as a biological syndrome, defined by a set of five specific symptoms: weakness; slowness; exhaustion; low physical activity; and shrinking (Fried et al., 2001). The frailty index is based on the cumulative deficit model, and covers non-specific diseases, deficits, signs, symptoms, disabilities, and mental factors (Rockwood and Mitnitski, 2007).

Notably, frailty and depression may share common risk factors and pathophysiologic pathways, including inflammation and mitochondrial dysfunction (Franceschi et al., 2018). As a result, frailty and depression also contribute to a range of harmful consequences of old age, such as poor quality of life, and increased health care needs, morbidity, and mortality (Rodda et al., 2011; Clegg et al., 2013; Shamliyan et al., 2013; Hare et al., 2014). Moreover, co-existing frailty and depressive symptoms have been reported to be associated with impaired cognitive functioning and disability based on a cross-sectional study from the Neurocognitive Outcomes of Depression in the Elderly study (Potter et al., 2016). To date, previous studies have demonstrated a strong link between frailty and depressive symptoms (Nabi et al., 2008; Surtees et al., 2008). As summarized by a meta-analysis based on 16 cross-sectional and 23 cohort studies in 2012, a positive association between depression and frailty was observed in cross-sectional studies, whereas findings from cohort studies were inconsistent (Mezuk et al., 2012). A 2017 meta-analysis from multiple countries reported that frail individuals have a 4.42-fold increased probability of being depressed and the likelihood of being frail is 4.07-fold higher in depressed patients (Soysal et al., 2017). Similarly, the increased likelihood of being frailty is 4.07-fold higher in depressed patients. In addition, the incidence of frailty among depressed patients is 2.72-fold higher than non-depressed patients, whereas the increased probability of being depressed was 0.90-fold higher in frail patients (Soysal et al., 2017). These findings suggest that frailty and depression may have an influence on each other, but no such data are available in China.

Currently, China has approximately 250 million old adults, accounting for 17.9% of the total 1.4 billion people (Jia et al., 2020). As expected, 27% of the population will be old populations by 2050 (United Nations, 2019). Hence, investigating the bidirectional association between frailty and depressive symptoms is older Chinese adults is warranted. To fill this gap in knowledge and provide the evidence, we conducted this study to analyze the bidirectional effect between frailty and depressive symptoms among older adults from the China Health and Retirement Longitudinal Study (CHARLS) using a cross-sectional and cohort design.

## Materials and Methods

### Study Participants

The study sample was obtained from the (CHARLS), a representative national cohort with middle-aged and older adults in China, as described previously (Zhao et al., 2014). At baseline, 17,708 participants from 450 urban and rural areas of 28 provinces were recruited in 2011, with follow-up evaluations in 2013, 2015, and 2018. During each survey, information on age, sex, marital status, educational level, family income, residence, social activities, retirement, cigarette smoking, alcohol consumption, sleep duration, number of chronic diseases, and body mass index (BMI) was collected from structured questionnaires and physical examinations by trained interviewers and physicians, respectively. Adults ≤ 60 years of age (*N* = 10,255), and those with missing depressive symptom assessments (*N* = 454) and demographic data (*N* = 50) were excluded. In addition, 1,646 or 1,361 participants without sufficient evaluation data on the frailty index or phenotypic frailty were excluded, respectively. Thus, using the baseline data in 2011, 5,303 or 5,117 participants underwent a cross-sectional analysis between frailty, as defined by a frailty index or phenotype, respectively, and depressive symptoms, using the baseline data in 2011. In the cohort study (2011–2018), we further excluded those participants with baseline frailty or depressive symptoms and those lost to follow-up. Therefore, 3,157 or 3,082 participants remained in the cohort analysis with baseline frailty, as defined by a frailty index or phenotype, respectively, and the incidence of depressive symptoms, while 2,086 or 1,491 participants were included for the cohort analysis between baseline depressive symptoms and incidence of frailty assessed by a frailty index or phenotype, respectively. Additionally, with the limited information, such as physical examination indicators, for the definition of phenotypic frailty in 2018, the association between baseline depressive symptoms and the incidence of phenotypic frailty was only evaluated during follow-up from 2011 to 2015. The flow chart of the selection process for participants is shown in Supplementary Figure 1.

This study was approved by the Peking University Ethical Committee. Informed consent was obtained from all participants.

### Assessment of Depressive Symptoms

The 10-item Center for Epidemiological Studies Depression Scale (CES-D) was used to evaluate depressive symptoms (Andresen et al., 1994). As reported elsewhere, the CES-D includes 10 items with four answers for each item, as follows: “rarely (<1 day/week);” “some days (1–2 days/week);” “occasionally (3–4 days/week);” “most (5–7 days/week).” For the negative items, the score was assigned 0, 1, 2, and 3 points for “rarely,” “some days,” “occasionally,” and “most,” respectively; whereas for the positive items, the score was defined as 3, 2, 1, and 0 points for “rarely,” “some days,” “occasionally,” and “most,” respectively. Then, the total score was summed for the 10 items, ranging from 0 to 30. In agreement with a prior study verified the validity of CES-D in CHARLS (Chen and Mui, 2014), the study participants were classified as depressed with a CES-D score ≥ 12 and non-depressed with a CES-D score < 12.

### Assessment of Frailty

Frailty was assessed by both phenotype and a frailty index. As reported elsewhere, phenotypic frailty was determined by the physical frailty phenotype (PEP) scale, which includes weakness, slowness, exhaustion, inactivity and weight loss (Wu et al., 2017). Weak was defined as a maximum handgrip strength for either hand less than the 20th percentile for the sex- and BMI- adjusted weighted population distribution. Slow was the average time of repeated walking tests over a 2.5-m course that exceeded the 80th percentile for the sex- and height- adjusted weighted population distribution. Exhaustion occurred when subjects felt that anything they did was an effort or they could not get going. Inactivity was defined as subjects who walked continuously for < 10 min in a typical week. Weight loss was defined as a self-rated loss of ≥5 kg in the previous year or a BMI ≤ 18.5 kg/m^2^. Using the above information, phenotypic frailty was categorized into three levels, as follows: robust (meeting none of the five domains); and prefrail and frail (meeting any one or more criteria).

The modified procedure of the China Kadoorie Biobank was used to evaluate the frailty index (Fan et al., 2020), and included 20 deficits, including chronic diseases (i.e., hypertension, heart disease, stroke, emphysema or bronchitis, tuberculosis, asthma, peptic ulcer, gallstone diseases, rheumatoid arthritis, fracture, neurasthenia, diabetes, cancer, and chronic kidney diseases), symptoms and signs (i.e., sleep disturbances, body pain or discomfort, unintentional weight loss, feeling sad or depressed, and poor health status), and physical measurements (i.e., BMI). Each deficit was dichotomized or mapped from 0 (the healthiest status) to 1 (the unhealthiest status). The frailty index was calculated as a ratio of the number of deficits for each participant to the total number of deficits, with a range from 0 to 1. Two subgroups were created, as follows: robust (≤0.10); and pre-frailty and frailty (>0.10). Because the number of frail people over 60 aged in the CHARLS is limited, we had to combine the prefrailty and frailty conditions.

### Statistical Analysis

The distributions of the study sample were determined by frailty status. Means ± standard deviations (SDs) or numbers (percentages) were calculated to perform continuous or categorical variables, respectively. Student’s *t*-test, the Mann-Whitney *U*-test and the chi-square test were used to compare the distribution of covariates in univariate analyses. Logistic regression models were used to determine the bidirectional associations between frailty and depressive symptoms with a cross-sectional design. Cox proportional hazard regression models were used to determine the relationship between frailty and the incidence of depression, as well as the relationship between depression and the incidence of frailty. For example, in the association between frailty index and the incidence of depressive symptoms, if individuals without depressive symptoms at baseline (2011), but were evaluated to be with depressive symptoms in 2013, then the time interval in Cox proportional hazards model was considered as 2 years. Similarly, if participant without depressive symptoms in 2011 and 2013, but were assessed to be with depressive symptoms in 2015, then the time interval was defined as 4 years; if individuals without depressive symptoms in 2011, 2013, and 2015, but were evaluated to be with depressive symptoms in 2018, then the time interval was considered as 7 years. Additionally, to ensure the reliability of the results, we further calculated the per SD change value after zero-mean normalization of the continuous independent variable. The standardization process was calculated according to the following formula:


$$z_{ij}=\left(x_{ij}-x_{i}\right)/s_{i}$$

where *z_ij_* represents the standardized independent variable, *x*_*ij*_ represents the original independent variable, *x_i_* represents the mean value of the independent variable, and *s_i_* represents the SD of the independent variable.

Moreover, subgroup analyses based on sex and residence were both performed to verify such associations, either in a cross-sectional or prospective study. In addition, a series of sensitivity analyses were further conducted. Firstly, because the definition of frailty might involve depressive symptoms-related factor (“Overall in the last month, how much of a problem did you have with feeling sad, low, or depressed?”), we excluded the associated factors and performed sensitivity analyses. Secondly, since the age limit established for old age is 65 years, we conducted the sensitivity analyses among people over the age of 65, as well. Thirdly, the survival time is unlikely to be precisely observed in the queuing setting, and thus at best it is known only to fall within an interval between two consecutive surveys, that is interval-censoring. Briefly, it treats the right-truncated observation value as a special interval with an infinite right boundary and the exact event time as a zero-length interval. The PROC ICLIFETEST (Guo et al., 2014) provides a non-parametric statistical method for estimating survival functions and a statistical test of interval-censored data. Therefore, we used this procedure to investigate the association between baseline frail status and incidence of depressive symptoms, as well as the association between baseline depressive symptoms and the incidence of frailty.

In the present study, the crude model was used to evaluate the odd ratio (OR) or hazard ratio (HR) and the 95% confidence interval (CI) without any adjustment. Model 1 adjusted for age and sex, and model 2 additionally adjusted for education level, smoking status, alcohol consumption, marital status, place of residence, income, participation in social activities, number of chronic diseases, retirement status, and sleep duration. All statistical analyses were conducted using SAS (version 9.4; SAS Institute Inc., Cary, NC, United States). All *P*-values were two-tailed, and a *P* < 0.05 was considered statistically significant.

## Results

### Basic Characteristics

Table 1 shows the basic characteristics of the study population. As calculated by the frailty index, greater than one-half of 5,303 participants (53.84%) were pre-frail or frail, primarily affecting the majority of women and retirees. These subjects tended to have a higher BMI, lower educational level and family income, shorter sleep duration, and several chronic diseases. In addition, the prevalence of depressive symptoms among pre-frail and frail subjects was significantly greater than the robust subjects. Indeed, 65.88% of subjects had pre-frailty and frailty when determined based on phenotype. Specifically, the pre-frail and frail subjects were more likely to be older, females, no alcohol consumption, countrymen, and retirees. Furthermore, the pre-frail and frail subjects tended to have several chronic diseases, but a lower BMI, educational and income levels, and shorter sleep duration. The prevalence of depressive symptoms in pre-frail and frail subjects was significantly greater than the robust subjects.

**TABLE 1:** The basic characteristic of the study population in the cross-sectional study.

Characteristics	Total	Frailty index		*P*-value	Total	Phenotypic frailty		*P*-value
		Robust	Pre-frailty/frailty			Robust	Pre-frailty/frailty	
N (%)	5,303	2,448 (46.16)	2,855 (53.84)		5,117	1,746 (34.12)	3,371 (65.88)	
Males (N, %)	2,717 (51.24)	1,343 (54.86)	1,374 (48.13)	<0.001	2,639 (51.57)	983 (56.30)	1,656 (49.12)	<0.001
Age (Mean ± SD, years)	67.70 ± 6.40	67.57 ± 0.13	67.81 ± 0.12	0.18	67.62 ± 6.36	66.26 ± 0.15	68.33 ± 0.11	<0.001
BMI (Mean ± SD, years)	22.93 ± 3.99	22.43 ± 0.08	23.37 ± 0.07	<0.001	22.92 ± 3.92	23.71 ± 0.09	22.51 ± 0.07	<0.001
Marital status (Married, N, %)	4,192 (79.05)	1,959 (80.02)	2,233 (78.21)	0.11	4,051 (79.17)	1,464 (83.85)	2,587 (76.74)	<0.001
Residence (Rural, N, %)	3,337 (62.93)	1,566 (63.97)	1,771 (62.03)	0.15	3,255 (63.61)	988 (56.59)	2,267 (67.25)	<0.001
Educational level (Illiterate, N, %)	1,887 (35.58)	865 (35.33)	1,022 (35.80)	<0.001	1,811 (35.39)	470 (26.92)	1,341 (39.78)	<0.001
Income (≥mean value, N, %)	415 (7.83)	233 (9.52)	182 (6.37)	<0.001	406 (7.93)	211 (12.08)	195 (5.78)	<0.001
Participation in social activities (Yes, N, %)	2,426 (45.75)	1,135 (46.36)	1,291 (45.22)	0.40	2,357 (46.06)	913 (52.29)	1,444 (42.84)	<0.001
Retired (Yes, N, %)	2,219 (41.84)	943 (38.52)	1,276 (44.69)	<0.001	2,091 (40.86)	669 (38.32)	1,422 (42.18)	0.01
Smoking status (N, %)				<0.001				0.09
Non-smoker	3,001 (56.59)	1,382 (56.45)	1,619 (56.71)		2,884 (56.36)	948 (54.30)	1,936 (57.43)	
Ex-smoker	650 (12.26)	242 (9.89)	408 (14.29)		626 (12.23)	220 (12.60)	406 (12.04)	
Current smoker	1,652 (31.15)	824 (33.66)	828 (29.00)		1,607 (31.41)	578 (33.10)	1,029 (30.53)	
Drinking status (N, %)				0.17				<0.001
Never	3,678 (69.36)	610 (24.92)	651 (22.80)		3,525 (68.89)	1,119 (64.09)	2,406 (71.37)	
<1 Time/month	1,261 (23.78)	170 (6.94)	194 (6.80)		1,238 (24.19)	486 (27.84)	752 (22.31)	
≥1 Time/month	364 (6.86)	1,668 (68.14)	2,010 (70.40)		354 (6.92)	141 (8.08)	213 (6.32)	
Sleep duration (Mean ± SD, hours/per day)	6.17 ± 2.00	6.46 ± 0.04	5.91 ± 0.04	<0.001	6.17 ± 1.99	6.41 ± 0.05	6.04 ± 0.03	<0.001
Number of chronic diseases (N, %)				<0.001				<0.001
0	1,329 (25.06)	1,187 (48.49)	142 (4.97)		1,298 (25.37)	530 (30.36)	768 (22.78)	
1	1,586 (29.91)	999 (40.81)	587 (20.56)		1,528 (29.86)	544 (31.16)	984 (29.19)	
≥2	2,388 (45.03)	262 (10.70)	2,126 (74.47)		2,291 (44.77)	672 (38.49)	1,619 (48.03)	
Frailty scores (Mean ± SD)	0.14 ± 0.09	0.06 ± 0.03	0.21 ± 0.07	<0.001	1.04 ± 0.97	0	1.57 ± 0.77	<0.001
Depressive symptoms (N, %)	1,712 (32.28)	469 (19.16)	1,243 (43.54)	<0.001	1,630 (31.85)	124 (7.10)	1,506 (44.68)	<0.001
Depressive symptoms score (Mean ± SD)	9.09 ± 6.45	7.07 ± 5.62	10.81 ± 6.61	<0.001	9.02 ± 6.43	5.10 ± 3.86	11.05 ± 6.55	<0.001

*BMI, body mass index; SD, standard deviation.*

### Bidirectional Relationship of Frailty With Depressive Symptoms in the Cross-Sectional Study

**FIGURE 1:**
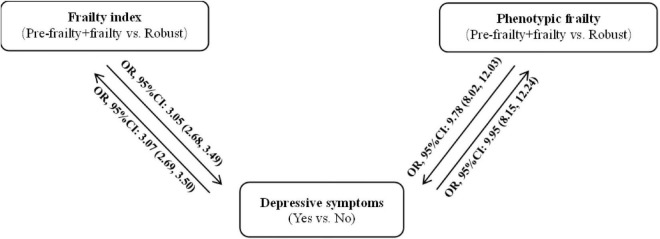
Bidirectional association between frailty and depressive symptoms in the cross-sectional study.

Figure 1 presents the association of frailty with depressive symptoms in the cross-sectional study. Pre-frail and frail participants had higher risks of depression before and after adjustments for confounders when compared to the robust participants. As shown in Supplementary Table 1, the crude ORs (95% CIs) for depressive symptoms among pre-frail and frail participants, as defined by the frailty index and phenotype, were 3.25 (2.87–3.69) and 10.56 (8.73–12.89), respectively. The ORs (95% CIs) for depressive symptoms for each incremental increase in the standard deviation of the frailty index and phenotypic frailty scores were 2.03 (1.91–2.17), and 2.58 (2.41–2.77), respectively. The ORs (95% CIs) for depressive symptoms among pre-frail and frail participants, as defined by the frailty index and phenotype were 3.19 (2.82–3.62) and 10.86 (8.95–13.29) after adjustment for age and sex, respectively. The ORs (95% CIs) for depressive symptoms for each incremental increase in the standard deviation of the frailty index and phenotypic frailty scores were 2.01 (1.88–2.14), and 2.76 (2.56–2.97), respectively. The full-adjusted ORs (95% CIs) for depressive symptoms among pre-frail and frail people defined by index and phenotype were 3.05 (2.68–3.49) and 9.78 (8.02–12.03), respectively. The ORs (95% CIs) for depressive symptoms for each incremental increase in the standard deviation of the frailty index and phenotypic frailty scores were 1.95 (1.82–2.08), and 2.61 (2.42–2.82), respectively.

When assigning depressive symptoms as an independent variable, as shown in Supplementary Table 2, depressed patients had higher risks of pre-frailty and frailty, with crude ORs (95% CIs) of 3.25 (2.87–3.69) for the frailty index and 10.56 (8.73–12.89) for phenotypic frailty. The ORs (95% CIs) for pre-frailty and frailty, as assessed by the frailty index and phenotype, for each incremental increase in the standard deviation of the depressive symptoms score were 1.90 (1.79–2.02) and 3.78 (3.46–4.16), respectively. After adjusting for age and sex, depressed patients had higher risks of pre-frailty and frailty, with ORs (95% CIs) of 3.19 (2.81–3.62) for the frailty index; and 10.88 (8.97–13.31) for phenotypic frailty. The ORs (95% CIs) for pre-frailty and frailty, as assessed by the frailty index and phenotype, for each incremental increase in the standard deviation of the depressive symptoms score were 1.89 (1.77–2.01) and 3.90 (3.55–4.29), respectively. Moreover, depressed patients had higher risks of pre-frailty and frailty, with ORs (95% CIs) of 3.07 (2.69–3.50) for the frailty index and 9.95 (8.15–12.24) for phenotypic frailty, after adjusting for age, sex, education level, smoking status, alcohol consumption, marital status, place of residence, income, participation in social activities, number of chronic diseases, retirement status, and sleep duration. The ORs (95% CIs) for pre-frailty and frailty, as assessed by the frailty index and phenotype, for each incremental increase in the standard deviation of the depressive symptoms scores were 1.89 (1.77–2.02) and 3.97 (3.59–4.40), respectively.

### Sensitivity Analysis Between Frailty and Depressive Symptoms in the Cross-Sectional Study

Because the definition of frailty might involve depressive symptom-related factors, sensitivity analysis was performed after excluding the related factors, as shown in Supplementary Table 3. The results were similar to that of the entire population. Pre-frail and frail participants had higher risks of depressive symptoms before and after adjustments for confounders, when compared to the robust participants. In the crude model, pre-frail and frail participants were at higher risk of depressive symptoms when assessed by the frailty index and phenotype [ORs (95% CIs), 3.46 (3.00–4.00) and 1.69 (1.51–1.91), respectively], compared to robust subjects. In addition, the ORs (95% CIs) for depressive symptoms per each incremental increase in the standard deviation for the frailty index and phenotypic frailty scores were 1.95 (1.83–2.08) and 1.32 (1.25–1.40), respectively. After adjusting for age and sex, pre-frail and frail participants were at higher risk of depressive symptoms when assessed by the frailty index and phenotype [ORs (95% CIs), 3.32 (2.87–3.84) and 1.73 (1.53–1.95), respectively], respectively, compared to robust subjects. In addition to the ORs (95% CIs) for depressive symptoms per each incremental increase in the standard deviation for the frailty index and phenotypic frailty scores were 1.93 (1.81–2.06), and 1.35 (1.27–1.43). In the full-adjusted model, pre-frail and frail participants were at higher risk of depressive symptoms when assessed by an index and phenotype [ORs (95% CIs), 3.12 (2.68–3.63) and 1.59 (1.40–1.82), respectively], compared to robust subjects. The ORs (95% CIs) for depressive symptoms per each incremental increase in the standard deviation for the frailty index and phenotypic frailty scores were 1.88 (1.76–2.01) and 1.28 (1.20–1.37), respectively.

When assigning depressive symptoms as an independent variable, as shown in Supplementary Table 4, depressed participants had a higher risk of pre-frailty and frailty before and after adjustments for confounding factors, including age, sex, education level, smoking status, alcohol consumption, marital status, place of residence, income, participation in social activities, number of chronic diseases, retirement status, and sleep duration, when compared to the non-depressed participants. In the crude model, the ORs (95% CIs) for pre-frailty and frailty, as defined by the frailty index and phenotype among depressed patients, were 3.46 (3.00–4.00) and 1.69 (1.51–1.91), respectively. The ORs (95% CIs) for pre-frailty and frailty, as assessed by the frailty index and phenotype, for each incremental increase in the standard deviation for the depressive symptoms scores were 1.98 (1.84–2.11) and 1.34 (1.27–1.42), respectively. After adjusting for age and sex, the ORs (95% CIs) for pre-frailty and frailty, as defined by the frailty index and phenotype among depressed patients, were 3.31 (2.87–3.84) and 1.73 (1.53–1.95), respectively. The ORs (95% CIs) for pre-frailty and frailty, as assessed by the frailty index and phenotype, for each incremental increase in the standard deviation for the depressive symptoms scores were 1.93 (1.80–2.07) and 1.36 (1.28–1.44), respectively. The full-adjusted ORs (95% CIs) for pre-frailty and frailty, as defined by the frailty index and phenotype among depressed patients were 3.15 (2.71–3.67) and 1.59 (1.40–1.82), respectively. The ORs (95% CIs) for pre-frailty and frailty, as assessed by the frailty index and phenotype, for each incremental increase in the standard deviation for the depressive symptoms scores were 1.92 (1.79–2.08) and 1.31 (1.23–1.40), respectively.

Consistently shown in Supplementary Figure 2, after adjusting for age, sex, education level, smoking status, alcohol consumption, marital status, place of residence, income, participation in social activities, number of chronic diseases, retirement status, and sleep duration, pre-frail and frail participants (>65 years) were at higher risk of depressive symptoms when assessed by an index and phenotype [ORs (95% CIs), 2.78 (2.35–3.30) and 11.03 (8.31–14.94), respectively], compared to robust subjects. In contrast, the full-adjusted ORs (95% CIs) for pre-frailty and frailty, as defined by the frailty index and phenotype among depressed patients were 2.79 (2.36–3.31) and 11.23 (8.45–15.22), respectively.

### Bidirectional Effect Between Frailty and Depressive Symptoms in the Cohort Study

The incidence of depressive symptoms was 56 per 1,000-person years. The association between frailty and the incidence of depressive symptoms is shown in Table 2. Compared with the robust participants, prefrail or frail participants had higher risks of depressive symptoms before and after adjusting for confounders, including age, sex, education level, smoking status, alcohol consumption, marital status, place of residence, income, participation in social activities, number of chronic diseases, retirement status, and sleep duration. The crude HRs (95% CIs) for depressive symptoms among prefrail and frail participants, as defined by the frailty index and phenotype, were 1.39 (1.23–1.58) and 1.38 (1.21–1.56), respectively. The HRs (95% CIs) for the incidence of depressive symptoms for each incremental increase in the standard deviation of the frailty index and phenotypic frailty scores were 1.24 (1.17–1.31), and 1.19 (1.12–1.26), respectively. After adjusting for age and sex, the HRs (95% CIs) for depressive symptoms among prefrail and frail participants, as defined by the frailty index and phenotype, were 1.38 (1.22–1.57) and 1.38 (1.21–1.57), respectively. The HRs (95% CIs) for the incidence of depressive symptoms for each incremental increase in the standard deviation of the frailty index and phenotypic frailty scores were 1.23 (1.16–1.30), and 1.19 (1.12–1.27), respectively. The full-adjusted HRs (95% CIs) for depressive symptoms among prefrail and frail people, as defined by an index and phenotype, were 1.38 (1.22–1.57), and 1.30 (1.14–1.48), respectively. The HRs (95% CIs) for the incidence of depressive symptoms for each incremental increase in the standard deviation of the frailty index and phenotypic frailty scores were 1.23 (1.16–1.30), and 1.15 (1.08–1.30), respectively.

**TABLE 2:** Association between frailty and incidence of depressive symptoms.

	HR (95% CI)		Per SD increase
	Robust	Pre-frailty/frailty	
**Frailty index**			
Unadjusted	1 (Reference)	1.39 (1.23–1.58)	1.24 (1.17–1.31)
Model 1	1 (Reference)	1.38 (1.22–1.57)	1.23 (1.16–1.30)
Model 2	1 (Reference)	1.38 (1.22–1.57)	1.23 (1.16–1.30)
**Phenotypic frailty**			
Unadjusted	1 (Reference)	1.38 (1.21–1.56)	1.19 (1.12–1.26)
Model 1	1 (Reference)	1.38 (1.21–1.57)	1.19 (1.12–1.27)
Model 2	1 (Reference)	1.30 (1.14–1.48)	1.15 (1.08–1.22)

*CI, confidence interval; HR, hazard ratio; SD, standard deviation. The crude model was conducted without any adjustment; Model 1 was adjusted for age, and sex; Model 2 was additionally adjusted for education level, smoking status, alcohol consumption, marital status, place of residence, income, participation in social activities, number of chronic diseases, retirement status, and sleep duration.*

When setting depressive symptoms as an independent variable, the incidences of frailty defined by phenotype and index were 45 per 1,000 person years and 94 per 1,000-person year. As shown in Table 3, depressed participants had a higher risk of pre-frailty and frailty when assessed by the frailty index, although the risk was not statistically significant. The crude HRs (95% CIs) for the incidence of pre-frailty and frailty, as defined by the frailty index and phenotype among depressed patients, were 1.17 (0.98–1.39) and 1.47 (0.95–2.28), respectively. The HRs (95% CIs) for the incidence of pre-frailty and frailty, as assessed by the frailty index and phenotype, for each incremental increase in the standard deviation of the depressive symptoms scores, were 1.06 (0.996–1.12) and 1.33 (1.17–1.53), respectively. With adjustment for age and sex, the adjusted HRs (95% CIs) for the incidence of pre-frailty and frailty, as defined by the frailty index and phenotype among depressed patients, were 1.15 (0.96–1.37), and 0.98 (0.63–1.52), respectively. The HRs (95% CIs) for the incidence of pre-frailty and frailty, as assessed by the frailty index and phenotype, for each incremental increase in the standard deviation of the depressive symptoms scores, were 1.05 (0.99–1.11) and 1.14 (0.995–1.31), respectively. Moreover, the full-adjusted HRs (95% CIs) for the incidence of pre-frailty and frailty, as defined by the frailty index and phenotype among depressed patients, were 1.18 (0.98–1.41) and 0.96 (0.61–1.50), respectively. The HRs (95% CIs) for the incidence of pre-frailty and frailty, as assessed by the frailty index and phenotype for each incremental increase in the standard deviation of the depressive symptoms scores were 1.06 (0.99–1.13) and 1.12 (0.96–1.29), respectively.

**TABLE 3:** Association between depressive symptoms and incidence of frailty.

	HR (95% CI)		Per SD increase
	Normal	Depression	
**Frailty index**			
Unadjusted	1 (Reference)	1.17 (0.98–1.39)	1.06 (0.996–1.12)
Model 1	1 (Reference)	1.15 (0.96–1.37)	1.05 (0.99–1.11)
Model 2	1 (Reference)	1.18 (0.98–1.41)	1.06 (0.99–1.13)
**Phenotypic frailty**			
Unadjusted	1 (Reference)	1.47 (0.95–2.28)	1.33 (1.17–1.53)
Model 1	1 (Reference)	0.98 (0.63–1.52)	1.14 (0.995–1.31)
Model 2	1 (Reference)	0.96 (0.61–1.50)	1.12 (0.96–1.29)

*CI, confidence interval; HR, hazard ratio; SD, standard deviation. The crude model was conducted without any adjustment; Model 1 was adjusted for age, and sex; Model 2 was additionally adjusted for education level, smoking status, alcohol consumption, marital status, place of residence, income, participation in social activities, number of chronic diseases, retirement status, and sleep duration.*

### Sensitivity Analysis Between Frailty and Depressive Symptoms in the Follow-Up Study

In agreement with the results from the entire study population, the sensitivity analysis showed that pre-frail or frail participants had a higher risk of depressive symptoms before and after adjustments for confounding factors, including age, sex, education level, smoking status, alcohol consumption, marital status, place of residence, income, participation in social activities, number of chronic diseases, retirement status, and sleep duration, when compared with the robust participants (Supplementary Table 5). The crude HRs (95% CIs) for depressive symptoms among pre-frail and frail participants, as assessed by the frailty index and phenotype, were 1.58 (1.39–1.81) and 1.17 (1.03–1.34), respectively. The depressive symptom HRs (95% CIs) for each incremental increase in standard deviation of the frailty index and phenotypic frailty scores were 1.22 (1.15–1.30) and 1.09 (1.03–1.16), respectively. After adjusting for age and sex, the HRs (95% CIs) for depressive symptoms among pre-frail and frail participants, as assessed by the frailty index and phenotype were 1.55 (1.36–1.77) and 1.19 (1.04–1.35), respectively. The depressive symptom HRs (95% CIs) for each incremental increase in standard deviation of the frailty index and phenotypic frailty scores were 1.21 (1.15–1.29) and 1.10 (1.03–1.17), respectively. The multivariate-adjusted HRs (95% CIs) for depressive symptoms among pre-frail and frail participants, as assessed by the frailty index and phenotype, were 1.55 (1.35–1.77) and 1.16 (1.02–1.32), respectively. The depressive symptoms HRs (95% CIs) for each incremental increase in standard deviation of the frailty index and phenotypic frailty scores were 1.22 (1.15–1.29) and 1.09 (1.02–1.16), respectively. When depressive symptoms were set as an independent variable (Supplementary Table 6), no significant relationship of existed between depressive symptoms and the incidence of frailty.

Using the ICLIFETEST procedure, the results were consistent with that of the main analysis. As shown in Supplementary Table 7, the crude HRs (95% CIs) for depressive symptoms among pre-frail and frail participants, as assessed by the frailty index and phenotype, were 1.44 (1.27–1.63) and 1.41 (1.24–1.60), respectively. With adjustment for age and sex, the HRs (95% CIs) for depressive symptoms among pre-frail and frail participants, as assessed by the frailty index and phenotype were 1.43 (1.26–1.62) and 1.42 (1.24–1.61), respectively. Moreover, the multivariate-adjusted HRs (95% CIs) for depressive symptoms among pre-frail and frail participants, as assessed by the frailty index and phenotype, were 1.44 (1.27–1.63) and 1.35 (1.19–1.54), respectively. However, considering the depressive symptoms as an independent variable, no significant relationship of existed between depressive symptoms and the incidence of frailty (Supplementary Table 8).

Moreover, for participants aged over 65, we got a similar result. As shown in Supplementary Figure 3, the multivariate-adjusted HRs (95% CIs) for depressive symptoms among pre-frail and frail participants (age over 65), as assessed by the frailty index and phenotype, were 1.25 (1.05–1.49) and 1.27 (1.06–1.53), respectively. Whereas considering depressive symptoms as an independent variable, the full-adjusted HRs (95% CIs) for the incidence of pre-frailty and frailty, as defined by the frailty index and phenotype among depressed patients, were 1.22 (1.00–1.49) and 0.92 (0.43–1.97), respectively.

### Subgroup Analysis Between Frailty and Depressive Symptoms in the Cohort Study

Tables 4, 5 present the subgroup analysis of the association between frailty and incidence of depressive symptoms, according to sex and place of residence. No significant interaction effects of frailty were detected with sex, or the place of residence. Pre-frailty and frailty participants, as assessed by the frailty index, had a higher risk of depressive symptoms [HRs (95% CIs), 1.27 (1.05–1.53) for males; 1.48 (1.24–1.76) for females; 1.53 (1.21–1.94) for urban residents; and 1.33 (1.14–1.55) for rural residents]. The HRs (95% CIs) for the incidence of depression for each incremental increase in the standard deviation for frailty index scores were 1.28 (1.15–1.42) for males, 1.28 (1.16–1.42) for females, 1.37 (1.20–1.56) for urban residents, and 1.25 (1.14–1.36) for rural residents. Pre-frail and frail participants assessed by phenotype had a higher risk of depressive symptoms [HRs (95% CIs), 1.45 (1.19–1.75) for males; 1.25 (1.05–1.50) for females; 1.35 (1.07–1.72) for urban residents; and 1.28 (1.10–1.50) for rural residents). The HRs (95% CIs) for the incidence of depressive symptoms for each incremental increase in the standard deviation for phenotypic frailty scores were 1.26 (1.13–1.41) for males, 1.16 (1.05–1.29) for females, 1.18 (1.02–1.36) for urban residents, and 1.18 (1.08–1.29) for rural residents. The results of subgroup analysis between depressive symptoms and the incidence of frailty were consistent with the entire population (data not shown). Thus, no significant interaction effects of depressive symptoms were detected with sex, or the place of residence.

**TABLE 4:** Subgroup analysis of the association between frailty and incidence of depressive symptoms according to the sex.

	HR (95% CI)		*P*_*interaction*_	Per SD increase	*P*_*interaction*_
	Robust	Pre-frailty/frailty			
**Frailty index**					
Males	1 (Reference)	1.27 (1.05–1.53)	0.25	1.28 (1.15–1.42)	0.71
Females	1 (Reference)	1.48 (1.24–1.76)		1.28 (1.16–1.42)	
**Phenotypic frailty**					
Males	1 (Reference)	1.45 (1.19–1.75)	0.17	1.26 (1.13–1.41)	0.87
Females	1 (Reference)	1.25 (1.05–1.50)		1.16 (1.05–1.29)	

*CI, confidence interval; HR, hazard ratio; SD, standard deviation. The model was adjusted for age, education level, smoking status, alcohol consumption, marital status, place of residence, income, participation in social activities, number of chronic diseases, retirement status, and sleep duration.*

**TABLE 5:** Subgroup analysis of the association between frailty and incidence of depressive symptoms according to the place of residence.

	HR (95% CI)		*P*_*interaction*_	Per SD increase	*P*_*interaction*_
	Robust	Pre-frailty/frailty			
**Frailty index**					
Urban	1 (reference)	1.53 (1.21–1.94)	0.37	1.37 (1.20–1.56)	0.36
Rural	1 (reference)	1.33 (1.14–1.55)		1.25 (1.14–1.36)	
**Phenotypic frailty**					
Urban	1 (reference)	1.35 (1.07–1.72)	0.65	1.18 (1.02–1.36)	0.80
Rural	1 (reference)	1.28 (1.10–1.50)		1.18 (1.08–1.29)	

*CI, confidence interval; HR, hazard ratio; SD, standard deviation. The model was adjusted for age, sex, education level, smoking status, alcohol consumption, marital status, income, participation in social activities, number of chronic diseases, retirement status, and sleep duration.*

## Discussion

The current cohort study first explored the bidirectional relationship between frailty and depressive symptoms in older Chinese adults. The cross-sectional study showed a bidirectional association between frailty and depressive symptoms, but the cohort study only reported that frailty was a risk predictor for subsequent depressive symptoms.

As previously reported, frailty is associated with increased mortality, hospitalization, falls, and admission to long-term care facilities, all of which may lead to disability or functional dependence, which in turn may contribute to the development of depressive symptoms (Woods et al., 2005; Hoogendijk et al., 2019). In agreement with our findings, Brown et al. (2020) reported that phenotypic frailty was related to depressive symptoms among 134 US residents. Similarly, an increased odds of depressive symptoms were shown in pre-frail [OR (95% CI), 3.82 (3.72–3.93)] and frail [OR (95% CI), 11.23 (10.89–11.58)] older Mexican adults (≥60 years of age) than non-frail older Mexican adults (Sánchez-García et al., 2014). Moreover, several prospective studies have shown that the presence of frailty at baseline predicts new-onset incident depressive symptoms (Feng et al., 2014; Collard et al., 2015; Makizako et al., 2015). In contrast, physical activity protects against depressive symptoms among older adults from south and southeast Asia (Kadariya et al., 2019). As a result, these findings suggest that frailty is a longitudinal predictor of depressive symptoms.

Depressive symptoms, in contrast, is often accompanied by a sedentary lifestyle, physical inactivity, poor social relationships, slow gait speed, risk of falls, weight loss, and malnutrition, thus making depressive symptoms a predictor of frailty, which in turn can exacerbate the typical emotional symptoms of depression, including sadness, anhedonia, and helplessness (Paulson and Lichtenberg, 2013; Soysal et al., 2017). Using a cross-sectional design, studies from Europe and the United States have also indicated that older people with depressive symptoms are more likely to be frail than those without depressive symptoms (Chang et al., 2010; Gurina et al., 2011; Jurschik et al., 2012; Collard et al., 2014; Lohman et al., 2014; Pegorari and Tavares, 2014; Sanchez-Garcia et al., 2014; Espinoza and Hazuda, 2015), whereas one study from northeast Brazil showed a negative association between depressive symptoms and frailty in community-dwelling older adults [OR (95% CI), 1.782 (0.820–3.870)] (Sousa et al., 2012). The discrepancy in findings may vary with different methods of assessing depressive symptoms and frailty, and the composition of the study population. Moreover, prospective studies from Australia, the United States, and six Latin American countries have shown that depressive symptoms might be an adverse consequence of frailty among older adults (Woods et al., 2005; Lakey et al., 2012; Lohman et al., 2014; Almeida et al., 2015; Prina et al., 2019). A study from the Rugao Longevity and Aging Study conducted in China investigated the association between depression symptoms and frailty among 1,168 Chinese adults > 70 years of age with a cross-sectional and 3-year follow-up analysis (Zhang et al., 2020). It was found that depressive symptoms are associated with the prevalence of pre-frailty and frailty. In addition, depressive symptoms were related to a 2.79-fold increased risk of the 3-year incidence of frailty. We also reported a positive relationship between depressive symptoms and frailty in the cross-sectional study, but no significant association existed between baseline depressive symptoms and incidence of frailty for phenotypic frailty or frailty index. The inconsistent findings may be partially attributed to ethnicity and population differences, as well as the assessment of frailty. Further studies with large sample are warranted, especially in China.

Frailty and depressive symptoms often independently reflect the physical and mental health of individuals. A strong link has been demonstrated between physical and mental health (Mezuk et al., 2012). In a sense, depressive symptoms is a sign of psychological frailty (Fitten, 2015). Depressive symptoms and physical frailty share several clinical characteristics, such as loss of energy, fatigue, poor sleep, and reduced interest, which may attribute to common risk factors and pathophysiologic pathways. Aging, is often accompanied by the appearance of chronic, sterile, low-grade inflammation, which may be involved in the pathogenesis of age-related diseases (Franceschi et al., 2018). Increased inflammatory cytokines are not only closely related to decreased muscle mass and strength, but also have a negative impact on central dopaminergic function, which may lead to fatigue, motor slowing, and depressive symptoms (Brown et al., 2016). Growing evidence has shown that higher levels of circulating inflammatory cytokines (C-reactive protein and IL-6) have been confirmed in pre-frailty and frailty participants (Soysal et al., 2016; Ma et al., 2018), as well as depressed patients (Howren et al., 2009; Valkanova et al., 2013). Moreover, mitochondrial dysfunction, which has been reported in several neurodegenerative diseases, including depressive symptoms, might be another explanation for the increased circulating cytokines (Bansal and Kuhad, 2016). Muscle biopsies obtained from depressed participants were been shown to have decreased ATP production. Impaired mitochondrial respiration in peripheral blood mononuclear cells has also been reported in depressed patients (Brown et al., 2016). Taken together, these markers have a strong relationship with frailty syndrome symptoms (Arauna et al., 2020).

This study had several strengths. First, the bidirectional longitudinal association between frailty and depressive symptoms we analyzed added the evidence in Chinese old adults, which helps identifying the sequence of physical and psychological frailty and thus provide insights into interventions for adverse health outcomes. Second, two definitions were used to evaluate frailty in the present study (phenotype and an index), which may reflect different dimensions of frailty. Third, because there may be an overlap between frailty and depressive symptoms, we further excluded those items and performed subgroup analyses. The findings were consistent with the entire population, suggesting the reliability of the results. Some limitations should be acknowledged. First, although depressive symptoms was assessed by a self-rated questionnaire (CES-D), the CES-D has been validated (Chen and Mui, 2014) and widely applied (Ni et al., 2017; Luo et al., 2018; Liu et al., 2020; Shao et al., 2021) in this cohort. Second, the frailty index items were modified based on the questionnaire because an evaluation can be performed if a questionnaire contains health variables and sufficient valid health variables (Searle et al., 2008). Third, due to the limited information (physical examination indicators) for the definition of phenotypic frailty in 2018, the association between baseline depressive symptoms and the incidence of phenotypic frailty was only evaluated in follow-up appointments from 2011 to 2015. Thus, whether the findings in our study can be extended to clinical practice remains to be explored. Moreover, the exclusion of a large number of adults makes the study sample may not be representative of Chinese adults. Prospective studies among Chinese adults are still warranted. Additionally, the time points of frailty or depressive symptoms incidents were only calculated by the time interval of follow-up, which may not be the exact time of points and makes the results misestimated, given that limited manpower and resources make it difficult to conduct more frequent follow-up investigations.

## Conclusion

A positive bidirectional association between depressive symptoms and frailty status has been demonstrate in older Chinese adults. More importantly, given that frailty is a risk factor for later depression, healthcare practitioners should be increasingly aware of the relationship, to avoid the co-existing conditions of frailty and depressive symptoms. Further researches are still warranted to clarify the causal relationship between these two conditions among Chinese adults.

## Data Availability Statement

The datasets presented in this study can be found in online repositories. The names of the repository/repositories and accession number(s) can be found below: http://charls.pku.edu.cn/index.html.

## Ethics Statement

The studies involving human participants were reviewed and approved by the Peking University’s Ethical Committees. The patients/participants provided their written informed consent to participate in this study.

## Author Contributions

LC: conceptualization, formal analysis, visualization, and writing – original draft. YZ, HL, and MS: writing – review and editing. YW: conceptualization, resources, writing – review and editing, supervision. YX: conceptualization, resources, writing – review and editing, supervision, funding acquisition. All authors contributed to the article and approved the submitted version.

## Conflict of Interest

The authors declare that the research was conducted in the absence of any commercial or financial relationships that could be construed as a potential conflict of interest.

## Publisher’s Note

All claims expressed in this article are solely those of the authors and do not necessarily represent those of their affiliated organizations, or those of the publisher, the editors and the reviewers. Any product that may be evaluated in this article, or claim that may be made by its manufacturer, is not guaranteed or endorsed by the publisher.
