# Supplementary material for: Bidirectional Longitudinal Study of Frailty and Depressive Symptoms Among Older Chinese Adults
Source: Front Aging Neurosci. 2022 Feb 10;14:791971. doi: 10.3389/fnagi.2022.791971 (PMC8866966; doi:10.3389/fnagi.2022.791971)
Supplement: Supplementary file 1 [file Data_Sheet_1.docx]

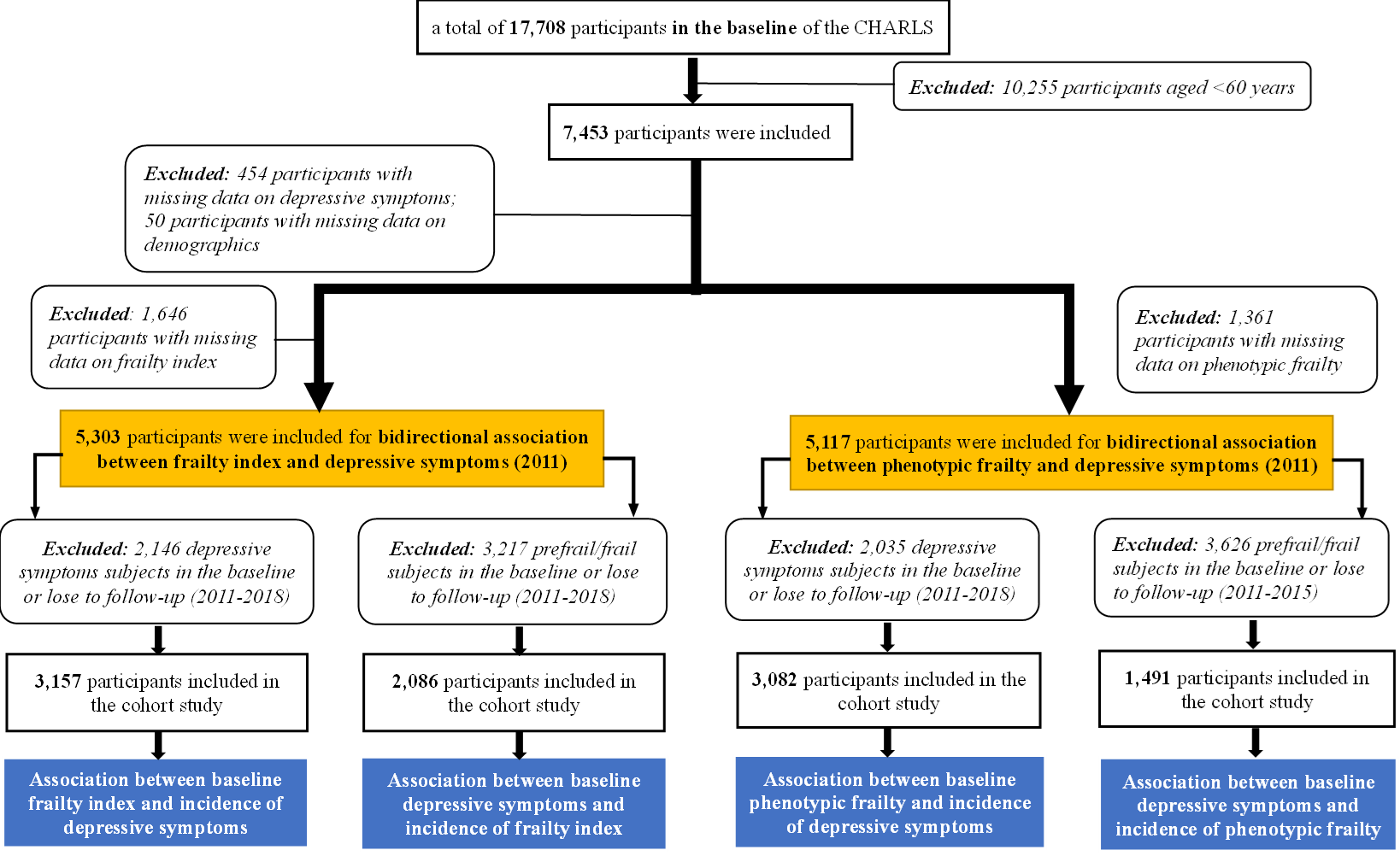
Supplementary Material

**Figure S1** Flow chart of the selection process of participants

**Table S1 Association between frailty and depressive symptoms in the cross-sectional study**

|  | OR (95% CI) | | per SD increase |
| --- | --- | --- | --- |
|  | Robust | Pre-frailty/Frailty |  |
| Frailty index |  |  |  |
| Unadjusted | 1 (reference) | **3.25 (2.87-3.69)** | **2.03 (1.91-2.17)** |
| Model 1 | 1 (reference) | **3.19 (2.82-3.62)** | **2.01 (1.88-2.14)** |
| Model 2 | 1 (reference) | **3.05 (2.68-3.49)** | **1.95 (1.82-2.08)** |
| Phenotypic frailty |  |  |  |
| Unadjusted | 1 (reference) | **10.56 (8.73-12.89)** | **2.58 (2.41-2.77)** |
| Model 1 | 1 (reference) | **10.86 (8.95-13.29)** | **2.76 (2.56-2.97)** |
| Model 2 | 1 (reference) | **9.78 (8.02-12.03)** | **2.61 (2.42-2.82)** |

CI, confidence interval; OR, odd ratio; SD, standard deviation.

The crude model was conducted without any adjustment; Model 1 was adjusted for age, and sex; Model 2 was additionally adjusted for education level, smoking status, alcohol consumption, marital status, place of residence, income, participation in social activities, number of chronic diseases, retirement status, and sleep duration.

**Table S2 Association between depressive symptoms and frailty in the cross-sectional study**

|  | OR (95% CI) | | per SD increase |
| --- | --- | --- | --- |
|  | Normal | Depressive symptoms |  |
| Frailty index |  |  |  |
| Unadjusted | 1 (reference) | **3.25 (2.87-3.69)** | **1.90 (1.79-2.02)** |
| Model 1 | 1 (reference) | **3.19 (2.81-3.62)** | **1.89 (1.77-2.01)** |
| Model 2 | 1 (reference) | **3.07 (2.69-3.50)** | **1.89 (1.77-2.02)** |
| Phenotypic frailty |  |  |  |
| Unadjusted | 1 (reference) | **10.56 (8.73-12.89)** | **3.78 (3.46-4.16)** |
| Model 1 | 1 (reference) | **10.88 (8.97-13.31)** | **3.90 (3.55-4.29)** |
| Model 2 | 1 (reference) | **9.95 (8.15-12.24)** | **3.97 (3.59-4.40)** |

CI, confidence interval; OR, odd ratio; SD, standard deviation.

The crude model was conducted without any adjustment; Model 1 was adjusted for age, and sex; Model 2 was additionally adjusted for education level, smoking status, alcohol consumption, marital status, place of residence, income, participation in social activities, number of chronic diseases, retirement status, and sleep duration.

**Table S3 Sensitivity analysis between frailty and depressive symptoms in a cross-sectional study after excluding the depressive symptoms-related factor**

|  | OR (95% CI) | | per SD increase |
| --- | --- | --- | --- |
|  | Robust | Pre-frailty/Frailty |  |
| Frailty index |  |  |  |
| Unadjusted | 1 (reference) | **3.46 (3.00-4.00)** | **1.95 (1.83-2.08)** |
| Model 1 | 1 (reference) | **3.32 (2.87-3.84)** | **1.93 (1.81-2.06)** |
| Model 2 | 1 (reference) | **3.12 (2.68-3.63)** | **1.88 (1.76-2.01)** |
| Phenotypic frailty |  |  |  |
| Unadjusted | 1 (reference) | **1.69 (1.51-1.91)** | **1.32 (1.25-1.40)** |
| Model 1 | 1 (reference) | **1.73 (1.53-1.95)** | **1.35 (1.27-1.43)** |
| Model 2 | 1 (reference) | **1.59 (1.40-1.82)** | **1.28 (1.20-1.37)** |

CI, confidence interval; OR, odd ratio; SD, standard deviation.

The crude model was conducted without any adjustment; Model 1 was adjusted for age, and sex; Model 2 was additionally adjusted for education level, smoking status, alcohol consumption, marital status, place of residence, income, participation in social activities, number of chronic diseases, retirement status, and sleep duration.

**Table S4 Sensitivity analysis between depressive symptoms and frailty in a cross-sectional study after excluding the depressive symptoms-related factor**

|  | OR (95% CI) | | per SD increase |
| --- | --- | --- | --- |
|  | Normal | Depressive symptoms |  |
| Frailty index |  |  |  |
| Unadjusted | 1 (reference) | **3.46 (3.00-4.00)** | **1.98 (1.84-2.11)** |
| Model 1 | 1 (reference) | **3.31 (2.87-3.84)** | **1.93 (1.80-2.07)** |
| Model 2 | 1 (reference) | **3.15 (2.71-3.67)** | **1.92 (1.79-2.08)** |
| Phenotypic frailty |  |  |  |
| Unadjusted | 1 (reference) | **1.69 (1.51-1.91)** | **1.34 (1.27-1.42)** |
| Model 1 | 1 (reference) | **1.73 (1.53-1.95)** | **1.36 (1.28-1.44)** |
| Model 2 | 1 (reference) | **1.59 (1.40-1.82)** | **1.31 (1.23-1.40)** |

CI, confidence interval; OR, odd ratio; SD, standard deviation.

The crude model was conducted without any adjustment; Model 1 was adjusted for age, and sex; Model 2 was additionally adjusted for education level, smoking status, alcohol consumption, marital status, place of residence, income, participation in social activities, number of chronic diseases, retirement status, and sleep duration.

**
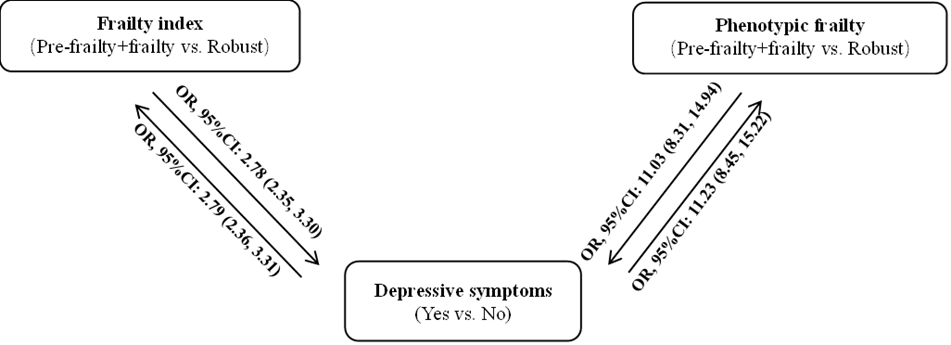
**

**Figure S2 Bidirectional association between frailty and depressive symptoms in the cross-sectional study among participants aged over 65**

**Table S5 Sensitivity analysis of the association between frailty and incidence of depressive symptoms after excluding the depressive symptoms-related factor**

|  | HR (95% CI) | | per SD increase |
| --- | --- | --- | --- |
|  | Robust | Pre-frailty/Frailty |  |
| Frailty index |  |  |  |
| Unadjusted | 1 (reference) | **1.58 (1.39-1.81)** | **1.22 (1.15-1.30)** |
| Model 1 | 1 (reference) | **1.55 (1.36-1.77)** | **1.21 (1.15-1.29)** |
| Model 2 | 1 (reference) | **1.55 (1.35-1.77)** | **1.22 (1.15-1.29)** |
| Phenotypic frailty |  |  |  |
| Unadjusted | 1 (reference) | **1.17 (1.03-1.34)** | **1.09 (1.03-1.16)** |
| Model 1 | 1 (reference) | **1.19 (1.04-1.35)** | **1.10 (1.03-1.17)** |
| Model 2 | 1 (reference) | **1.16 (1.02-1.32)** | **1.09 (1.02-1.16)** |

CI, confidence interval; HR, hazard ratio; SD, standard deviation.

The crude model was conducted without any adjustment; Model 1 was adjusted for age, and sex; Model 2 was additionally adjusted for education level, smoking status, alcohol consumption, marital status, place of residence, income, participation in social activities, number of chronic diseases, retirement status, and sleep duration.

**Table S6 Sensitivity analysis of the association between depressive symptoms and incidence of frailty after excluding the depressive symptoms-related factor**

|  | HR (95% CI) | | per SD increase |
| --- | --- | --- | --- |
|  | Normal | Depressive symptoms |  |
| Frailty index |  |  |  |
| Unadjusted | 1 (reference) | 1.12 (0.63-1.99) | 1.02 (0.84-1.24) |
| Model 1 | 1 (reference) | 1.06 (0.59-1.92） | 1.01 (0.83-1.23) |
| Model 2 | 1 (reference) | 0.98 (0.52-1.84) | 0.96 (0.78-1.19) |
| Phenotypic frailty |  |  |  |
| Unadjusted | 1 (reference) | 1.00 (0.91-1.10) | 1.001 (0.96-1.04) |
| Model 1 | 1 (reference) | 1.05 (0.96-1.16) | 1.03 (0.98-1.07) |
| Model 2 | 1 (reference) | 1.05 (0.95-1.16) | 1.02 (0.98-1.07) |

CI, confidence interval; HR, hazard ratio; SD, standard deviation.

The crude model was conducted without any adjustment; Model 1 was adjusted for age, and sex; Model 2 was additionally adjusted for education level, smoking status, alcohol consumption, marital status, place of residence, income, participation in social activities, number of chronic diseases, retirement status, and sleep duration.

**Table S7 Sensitivity analysis of the association between frailty and incidence of depressive symptoms using the ICLIFETEST procedure**

|  | HR (95% CI) | |
| --- | --- | --- |
|  | Robust | Pre-frailty/Frailty |
| Frailty index |  |  |
| Unadjusted | 1 (reference) | **1.44 (1.27-1.63)** |
| Model 1 | 1 (reference) | **1.43 (1.26-1.62)** |
| Model 2 | 1 (reference) | **1.44 (1.27-1.63)** |
| Phenotypic frailty |  |  |
| Unadjusted | 1 (reference) | **1.41 (1.24-1.60)** |
| Model 1 | 1 (reference) | **1.42 (1.24-1.61)** |
| Model 2 | 1 (reference) | **1.35 (1.19-1.54)** |

CI, confidence interval; HR, hazard ratio.

The crude model was conducted without any adjustment; Model 1 was adjusted for age, and sex; Model 2 was additionally adjusted for education level, smoking status, alcohol consumption, marital status, place of residence, income, participation in social activities, number of chronic diseases, retirement status, and sleep duration.

**Table S8 Sensitivity analysis of the association between depressive symptoms and incidence of frailty using the ICLIFETEST procedure**

|  | HR (95% CI) | |
| --- | --- | --- |
|  | Normal | Depressive symptoms |
| Frailty index |  |  |
| Unadjusted | 1 (reference) | **1.21 (1.01-1.44)** |
| Model 1 | 1 (reference) | 1.18 (0.99-1.41) |
| Model 2 | 1 (reference) | 1.21 (1.00-1.46) |
| Phenotypic frailty |  |  |
| Unadjusted | 1 (reference) | 1.45 (0.85-2.47) |
| Model 1 | 1 (reference) | 1.45 (0.86-2.47) |
| Model 2 | 1 (reference) | 1.38 (0.80-2.41) |

CI, confidence interval; HR, hazard ratio.

The crude model was conducted without any adjustment; Model 1 was adjusted for age, and sex; Model 2 was additionally adjusted for education level, smoking status, alcohol consumption, marital status, place of residence, income, participation in social activities, number of chronic diseases, retirement status, and sleep duration.

**
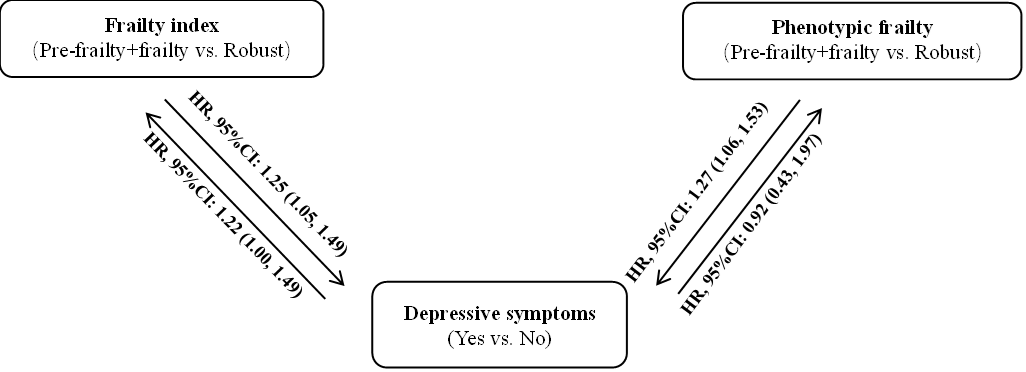
Figure S3** **Bidirectional association between frailty and depressive symptoms in the cohort study among participants aged over 65**
